# Foreign Summary

**Published:** 1840-05-02

**Authors:** 


### FOREIGN SUMMARY.

### PHILLIPS' LECTURE ON THE PRINCIPLES AND PRACTICE OF SURGERY.—NO. III.

CANCER—(Continued.)

*Medullary Form—Anatomical Characters—Chemical Characters—Distinctions between Scirrhoid and Medullary Tissues—Cutaneous Cancer—Formation—Carmichael's, Adams', Cruveilhier's, Müller's, Broussais', Carswell's Opinions—Period of Life—Causes—Diagnosis—Prognosis—Exceptions—Treatment.*

IN examining the progress of medullary structures, we may admit three stages; in the first, the tumour has the consistency of conglobate glands; in the second, it is softer; in the third, the softening is complete—it is almost semi-fluid, giving to the touch a sensation of fluctuation. A fourth period may be admitted; it is that of ulceration of the integuments, when the tumour is near the surface. The dissection of medullary structures (says Lobstein) shows that the mass is composed of three distinct parts—cells, parenchyma, and extravasated blood. The cellular tissue, which constitutes the frame-work of this structure, occupies only a very small space; it is soft and semi-transparent. The parenchyma is presented with different degrees of consistency: in the crude period it is more or less transparent, hard, divided into lobules; in the second period, the mass is like the brain of a young child, divided into lobules by fissures, in which blood vessels, whose texture is extremely delicate, are lodged; in the third period (that of perfect softening,) the consistency of the mass is not much greater than that of thick pus. Between the first and second periods, medullary structures present an appearance which has been little noticed. The consistency being considerable, when the tumour is incised a milky matter is yielded, not upon compression, but upon scraping the surface with the knife, though no cells or vesicles which could have contained it appear.

Berard injected an encephaloid tumour, and found that in its substance there was a great preponderance of arterial canals, and that this preponderance was as much greater as the softening was more decided. He found that the membranous envelope presented an abundant venous plexus, interlacing with the arterial ramifications. In a section of the tumour he could not see *a single venule or black point*, whilst the injection freely pervaded the adjoining thyroid body. Either, therefore, there were no veins, or they were filled up with some matter other than fluid blood; and, in fact, it was found, upon squeezing the mass, that encephaloid matter oozed out from numerous canals apparently venous. Velpeau describes a case in which the kidney was the seat of disease, and cancerous matter was found in the inferior cava. Many similar cases are detailed by Cruveilheir and others.

It is probable that the reason of the apparent absence of veins is this: the blood moves more slowly in the veins than in the arteries; it is in the veins that the blood becomes stagnated; the blood moving in the veins in a medullary tumour, while in this almost stagnant condition, may be transformed into a medullary mass, and the veins be, in this way, made impervious to injection. This explanation is not, perhaps, applicable to all cases, for in those of Berard there was destruction of the venous parietes; but whether the medullary matter found its way into the veins, or whether it was already transformed in the canal, is a matter which cannot easily be determined. In many specimens of medullary tissue, Cruveilhier thought the structure represented a sort of "feutre" or felt, or cavernous erectile tissue; that there was identity of aspect between the areolar frame-work of the cancerous and erectile tissue: this he thinks we cannot be surprised at, since the accidental erectile tissue is nothing else than a development of the venous capillary system; so that there is not only identity of aspect but of affinity between the erectile and cancerous tissue. "I regard," says he, "as a truth acquired in science, that cancer has its immediate seat in the venous system. A second variety is, says he, eminently vascular, appearing to be composed of veins flattened and flexuous, dilated into ampulla with extremely thin parietes, easily destroyed, and becoming the source either of small or large extravasations of blood. These are more rapid in their course than the hard varieties.

*Chemically.*—Some experiments were made a few years ago, in the laboratory of the Faculty of Medicine of Paris, upon cerebriform tissue in its first or crude stage. Treated first by cold water, it furnished a little albumen and gelatine; treated by warm water, the filtered liquid was turbid. When evaporated, it yielded gelatine and a little phosphate of lime; by means of warm alcohol a little more gelatine was obtained, but the greater part of the matter remained insoluble in water and alcohol, presenting a fibrous appearance, not unlike fibrin or gluten. Acetic acid produced considerable tumefaction of the residue. Cerebriform matter in its second stage was treated as follows:—Two hundred parts mixed with one hundred parts of distilled water formed at first a homogeneous mass; heated, it deposited a solid coagulated matter, insoluble in water, spongy in appearance, but tenacious, elastic, horny. This same coagulum, weighing a hundred and ninety-six 'fraumes,' burnt upon lighted charcoal, exhaled a smell like burnt horn, bearing the characters of coagulated albumen. The remaining water having been evaporated, no gelatinous appearance was assumed. From these experiments it resulted, that, at its first stage, it is richer in gelatine, whilst in its second stage it contains more albumen.

*Distinctions.*—The following are the prominent distinctions between these two classes of structures: at its perfect state of development the medullary disease presents a milky white pulpy matter, the surface studded here and there with red spots; scirrhus presents a lard-like mass, intersected by dense white bands. The medullary tissue presents many small arteries, becoming still more numerous where softening is decided; in some points there is actual extravasation, and when ulceration takes place, hæmorrhage follows. Scirrhus has very few vessels, extravasation is very unfrequent, and when ulceration occurs, hæmorrhage seldom happens. Medullary tissue often breaks down the walls of veins, and may get into the canal, which it may distend; we have no instances of scirrhus following a similar course. Medullary tissue may affect all organs; the seat of scirrhus is much more limited. Medullary tissue may acquire great bulk, and when it softens, the mass becomes elastic. Scirrhus rarely acquires great bulk, sometimes seems to produce atrophy of parts, is not well rounded nor elastic. Medullary matter may exist in many organs at the same time; scirrhus is generally confined to one point. Medullary tumours may be developed at an early period of life; scirrhus is rarely seen before adult age. Medullary tumours may be long in contact with the skin without adhering; scirrhus is soon adherent. Medullary tumours ulcerate very rapidly, destroying life often in a few weeks; scirrhous ulceration frequently continues for months, or even years.

*Cutaneous Cancer.*—There is a variety of cancer which commences in the skin. A tubercle or wart is usually presented. Scarpa examined the tissue of these tubercles under a lens, and ascertained that the tissue possessed the characters of scirrhus, but we shall in many cases vainly look for the scirrhous base which is usually found in scirrhoid ulcers; besides this, they are usually very long in affecting the neighbouring glands, and are often completely cured by extirpation. These cancers take a different course from those we have been considering: they commence with an ulcer, and gradually acquire a scirrhous fundus. They are most frequently developed about the head and face, about the areola of the nipple, the scrotum, the vulva, the rectum.

*Formation.*—Of the mode of formation of carcinomatous structures, we are profoundly ignorant. Carmichael believed it to be a living being, enjoying independent existence. Adams' opinion was, that the essence of cancer resided in the presence of a hydatiform animal, which he called hydatis carcinomatoso. According to him the white septa which intersect, and the membrane which surrounds scirrhus, are liviug cysts—tænia hydatoidea. The proof that this hydatid was living, is, that it was like those of sheep; that it shrivels and takes a granular appearance when an incision is made through the tissue, and that this does not happen in some hours after removal, when the breast is cold. He believed there were three kinds, serous, gelatinous, and sanguinolent. Hodgkin sought to prove that the presence of a serous membrane, having a cystiform arrangement, is necessary for the production of cancer; the existence of the former, he believes, precedes the latter. The objection which must be made to this view is, that it is too exclusive; there are certain carcinomatous structures in which a cystiform character may be shown; there are others in which it is impossible to demonstrate any thing of the kind. The cancer areolaire of Cruveilhier is "essentially constituted by the tranformation of the affected tissue into an areolar fibrous structure, filled by a kind of transparent gelatinous matter." He thinks it makes no exception to what he regards as a general law, "that all organic transformations are exclusively the result of a successive deposition of morbid products in the cellular element of organs." He believes it is rarely manifested, successively or simultaneously at a great number of points, whilst Müller believes that this areolar disease is ordinarily accompanied by general affection of the system, and that the patient dies with similar visceral disease. Cruveilhier believes it to be developed most frequently in bones, in the rectum, the uterus, the ovaries, the cœcum, the small intestine, but no where so frequently as in the stomach. Müller's opinion of the cystiform or cell-like arrangement of this form of cancer is somewhat different:—if we examine a very thin slice of this matter with a power equal to about 400 diameter, we distinguish a granular matter associated with cells or cysts; these cells are not single, but within the first we see a second, with which the first seems to have no direct cennection, "like one pill-box within another." The smaller cell contains a dark yellow parietal nucleus, which seems in contact within, perhaps arising from one side of the cell. He believes these cells, or cysts, arise from the granules; that they enlarge, burst, and are succeeded by others, and that in this way the mass increases; he applies, in fact, the opinion of Schleiden. According to him, each cell in vegetable developement contains a portion of starch, which is capable of being converted into nutritious matter. This conversion having taken place, a dark spot is perceived in the coats of the cell, and from this spot a new cell is seen to be protruded; accordingly, this spot being the germ of the cell, it is called by Schleiden the cytoblast. The new cell, when generated, gives birth in its interior to new cytoblasts, which again generate new cells, and thus a series of cells is produced, one within another, until the external one is ruptured, and its contents are enabled to escape, and thus to obtain their natural developement. The newly formed cells are of extreme tenuity, but new matter is afterwards deposited within the interstices of that originally formed, until they gradually acquire firmness and consistency. Without knowing any thing of this discovery, Mr. Gulliver showed me this arrangement in a cancer of the breast, removed from a patient of fifty-five, by my colleague, Mr, Stafford. He found considerable difficulty in determining whether the granular masses succeeded to the cysts, or *vice versâ*, though he inclined to the former opinion. I have examined many other cases, but without discovering a similar arrangement. The patient in Mr. S.'s case died of pneumonia, and no similar deposites were found in any of the viscera. Mr. Gulliver's notes on the structure in this case, are as follows:—"Ultimate structure of juice, molecular; globules very variable in size; many oil-like, floating freely in fluid; many contained in transparent cysts of pretty large size, very variable in shape: some oval, others globular; molecules and cysts contained in an interlacement of cellular tissue. Certainly one cyst inclosing others. After maceration in muriate of soda, one oval cell seen to enclose two others with a nucleus (intervening parts granular;) length of large cyst 1-400th of an inch; breadth 1-555th; circular nuclei 1-5333d." Mr. Gulliver is about to submit to examination a large number of specimens of cancer, from which he expects it will result that this cellular form will be very prevalent.

Broussais and his followers believe that cancer is a consequence of inflammatory action; "all inflammatory and sub-inflammatory action may determine cancer." The authors of the Dict. de Med. art. Cancer (Breschet and Ferrus,) maintain that cancer succeeds to irritation or inflammation, and cannot be developed without being preceded by them. This irritation or inflammation determines the deposite of coagulable lymph, which may harden and constitute the nucleus of scirrhus; inflammation may attack this nucleus and disorganise it; but in its passage from an organic to an inorganic state, there will be a detritus from which cerebriform matter results; if it be mixed with blood, or new capillaries be formed, fungus hæmatodes may result. In this theory two distinct inflammatory actions are required or invoked; the one primitive, which may be only inflammatory irritation, but determines the formation of scirrhus; the other secondary, by which it is changed into medullary matter. Of this, I am not aware that any proof can be given; we are not therefore justified in assuming that scirrhus results from inflammatory irritation. In fact do we find scirrhus oftenest where irritation is most decided? Is it in abandoned women that cancer of the uterus is most frequently seen? Is cancer of the mammary gland more common in those women who have nursed many times than in those who have never had children to nurse? I believe not. If we examine scirrhus soon after it is detected, we do not find the smallest vestige of inflammation, yet it may increase. Now, if it can enlarge without inflammation, may it not be formed without it? In as far as concerns the second degree of scirrhus, or its passage into the period of softening, it seems to me that an irritant action must be admitted. What else than inflammatory irritation could produce the change of texture?

The following reasons seem to me conclusive against these tissues being a consequence of inflammation: that they are manifested by characters essentially different from those of inflammation; that they are not necessarily preceded by inflammation; that inflammation does not explain the accidents they determine, nor the disorders which characterize them; that they never seem to arise simply under the influence of causes of inflammation, and are not manifested by the same symptoms; that they are preceded by the formation of an organized tissue, whose nature and aspect is different from that of tissues which have been altered by inflammatory action; that the formation of this tissue, either in masses or infiltrated in the cells of organs, is explained, naturally and simply, by an alteration in nutrition, which is characterized by the secretion into the diseased part of a substance which is at first organized and afterwards softened, disorganized, and, at a certain period of its existence, reduced to a pulpy or detritic mass; that scirrhus and cerebriform matter is identical, or nearly so, in all tissues, whilst inflammations are not perfectly the same in all tissues. Inflammation being a disease of tissues, is modified by their vitality and their organization; whilst cancer, resulting from the primitive formation of a morbid substance which constitutes it, is never primarily subjected to the particular state of the organ in which it is developed or deposited.

Dr. Carswell says "that this substance (cancerous matter) exists in the blood." That the deposit is a consequence of a modification of the blood, is evident from the particular mode of deposition; from the existence of this matter in the vessels ramifying in carcinomatous matter; from its being found in vessels having no direct communicution with an organ affected with cancer, and in blood which has been effused into the cellular tissue or upon the surface of organs. The divisions of the vascular system in which it has been found are the venous and the capillary. Whether it be, as Dr. Carswell observes, "that the presence of an organized product in the blood can have no other origin than the blood itself, and that it cannot be introduced into this fluid by absorption; whether it be a product of secretion, and not existing in the blood; or whether, as in the two cases described by Berard, the matter got into veins passing through the carcinomatous mass by a destruction of their parietes, may be a matter of question. Velpeau thinks that the blood in a vein may be converted into carcinomatous matter. Cruveilhier, at the same time that he believes the cellular tissue to be the structure in which cancerous matter is deposited, expresses his conviction that all new products, all morbid alterations, are formed at the expense of the venous capillary system. Laennec says it is a lesion of structure; Cruveilhier, Andral, Lobstein, and Carswell, that it is a lesion of secretion of nutrition. There are, says Carswell, several organs where means are afforded for ascertaining the seat, origin, and mode of formation of cancer; but it is necessary to observe it at an early period of its existence. Investigated in this its first stage, we ascertain, with greater or less facility, that this substance becomes manifest to our senses, either as a production of nutrition or of secretion. In the former case it is deposited in the same manner as the nutritive element of the blood, enters into the molecular structure, and assumes the form and arrangement of the tissue or organ into which it is introduced; in the latter it makes its appearance on a free surface, after the manner of natural secretions, as on serous surfaces in general. It may be found not only in the molecular structure, and on the free surface of organs, but also in the blood. He believes that an organ is often not at all enlarged by the deposition of carcinomatous matter, and that it is a mere exchange for an equal quantity of the natural tissue, which has been absorbed in the usual way.

*Period of Developement.*—Although what is termed the critical period of life, or that included between 35 and 55, be the time of life when carcinoma is most frequently developed, it may also occur earlier or later. In the earlier periods of life, the variety usually seen is medullary; it may affect the eye or it may affect the skin. A few months ago, I had a cancer of the perineum, in a young woman of 23. I have at present, cancer of the rectum, in a lad of 17. Not long since, a young woman died, at the age of 21, from cancer of the uterus. It is certain that women suffer from the disease more frequently than men.

*Causes.*—Of the cause of cancer we are utterly ignorant. We have no evidence to show that cancer is contagious; cancerous ichor has often been inoculated, but I am not aware that it has ever produced cancer. Men have cohabited with woman suffering from cancer of the neck of the uterus, but without propagating the disease. Neither have we any evidence to prove that it is hereditary; though the popular opinion favours that belief. And again, with regard to the depressing passions, also assigned as a cause, we see cancer developed in persons habitually cheerful, in whom no function has been seriously injured, and who have enjoyed good health; and in whom no external injury has been inflicted upon the organ which is the seat of the disease. With respect to the cessation of menstruation, we cannot deny that a tumour, long indolent, may rapidly increase under it; but, on the other hand, how frequently do we see cancers even in a state of ulceration before the cessation of this function, and even while it is regularly performed.

Some persons believe that a mysterious unknown condition, termed *diathesis*, excites the development of cancer; they believe that there exists, in certain cases, an internal disposition sufficient to produce cancer; it is, say they, the true and unique cause of the reproduction of cancer after extirpation; it is upon it that the simultaneous or successive development of the disease in several organs, far removed from each other, depends. According to them, this diathesis may exist many years, or the whole of life, without being manifested by any external or internal sign. Bayle goes so far as to say that cancer is never a local disease, though determined by an external cause; that it is to this diathesis that cancers owe the property of reproduction twenty years after extirpation, though, during the intermediate time, the patient has seemed to enjoy good health.

To consider, as the reproduction of extirpated cancer, that which is manifested twenty years afterwards, whilst, during this long interval, the patient has had seemingly good health, is too great a stretch for my imagination. It establishes incontestibly, if admitted, that cancer is an incurable disease.

That cancer is a disease whose local manifestation is secondary, is highly probable; thus twenty women, at the age of 45, placed under apparently exactly similar circumstances, shall receive a similar blow on the breast; in one case cancer may succeed to the injury—contusion happens in all, but the one has a disposition to the disease, and the disposition is excited into action by the injury. There are certain "constitutional" diseases which we know how to excite—syphilis and scorbutus; there are others which we know not how to excite at will—phthisis and cancer.

*Diagnosis.*—With all the knowledge we can bring to bear upon the subject of cancer, the diagnosis is still in many cases a matter of great difficulty. When the tumour is indolent, and ulceration has not taken place, it is often impossible to pronounce upon it with any certainty. Though accessible to the eye and the touch, and not very deep seated, we frequently cannot positively determine, before extirpation or puncture, the morbid structure of which it is composed: neither the length of time it has existed, nor the obscurity of the causes by which it has been produced, nor its density, nor its mobility, furnish certain indications upon which to determine whether the tumour be formed of scirrhus, or medullary, or fibrous or fibro-cartilaginous matter, or whether it results from a simple chronic induration of the affected organ. But if our power of diagnosis be not sufficiently precise to make out these anatomical peculiarities, we can in most instances distinguish, from all others, scirrhus and cancerous productions, and bring to bear a sufficient number of circumstances to justify an operation. Scirrhus tumours, externally situated, may be confounded with all others which exist without heat, change of colour, and fluctuation. The presence of one of these signs suffices to exclude all idea of scirrhous or unsoftened medullary matter, and acute lancinating pains, occurring with irregular intervals, can rarely be confounded with that determined by ordinary inflammation. The situation of aneurisms in the course of arteries, and their pulsation isochronous with the motion of the heart, suffice even when they are firm and not fluctuating, to distinguish them from scirrhus. Fatty and encysted tumours present either an inelastic softness, or an obscure and imperfect fluctuation, which distinguishes them from organic products, and they are commonly not developed in those situations where scirrhus is usually found. Certain dense fibrous or fibro-cartilaginous cysts, containing either hydratids or suetty matter, may sometimes mislead, but then such tumours are not common externally, and they always present an elastic resistance, and usually an obscure fluctuation, by means of which we might distinguish their true character. It is generally more difficult to distinguish scirrhus from fibrous tumours, which are not very unfrequently developed in the mamma, and are often found in the uterus. But these fibrous tumours have generally a smooth surface, rounded and regular forms, neatly separated from adjoining tissues, which contrast with the knotted surfaces, close adhesion, and great density and weight of scirrhus. If a tumour remain long, hard, and apparently inert; gradually becomes the seat of lancinations, which increase in frequency; if soft, and apparently fluctuating points appear, under which the skin is thinned and reddened, there can be no doubt that malignant disease exists. It may happen that a tumour exists in the breast, and that a sanious fluid escapes from the nipple. This very grave symptom may however mislead; I have had the history of two cases in which the tumour of the breast had succeeded to a blow; after a time a sanious fluid flowed from the nipple, the tumour was extirpated, and was found to be formed by a clot of blood succeeding to the injury: the clot broke down, and its more fluid portion found its way into the lactiferous ducts. Even when ulceration has occurred, when we see an ulcer with a scirrhous base, everted edges, ichorous, fetid, acrid secretion, with a tendency to hæmorrhage, these circumstances are not peculiar to each species of cancer. Cancer of the face is often altogether indolent, and the scirrhous base is often not found elsewhere than in cancer of glandular structures. The everted cut edges, the tendency to hæmorrhage, fungous vegetation, are seen in ulcers which are not cancerous, and all cancerous ulcers do not yield a fetid sanies; therefore these so called characteristic signs have only a relative value. The specific odour of the pus, it is important to notice, the eroding character of the ulcer, the tendency to swell of the neighbouring glands, and the appearance of suffering impressed upon the countenance, the pale, dry, parchment-like skin, and the tendency to reproduction. In spite of all these signs, it must be borne in mind that, in an affection the proximate cause of which is unknown, there can be little certainty.

*Prognosis.*—Can cancer be cured? I believe there can be no question that a cancerous tumour is not susceptible of resolution; we know no medical agent capable of eradicating it from the constitution. We do not think it impossible that ultimately such means may be discovered. We have a panacea for syphilis; we may, by change of climate, now and then arrest the progress of tubercles. With regard to cancer, its progress has been arrested; and after ablation in some cases, few though they be, the disease has not reappeared; if this be so, we must admit that the cause under the influence of which it was principally developed has been destroyed or arrested in the interval. The cases described by Mr. Hill, eighty-eight in number, I do not propose, to consider, because they are so opposite to the results of general experience, but of these eighty-eight only five were tumours of the breast, and of these five only two were apparently cured. Those of Recamier are to me little more satisfactory. Monro operated, by ablation, in sixty cases of cancer; at the end of two years four only had not suffered relapse. Scarpa practised his profession very extensively for sixty years, and only knew three cases in which the disease had not reappeared. Boyer removed a hundred cancerous tumours; in four or five cases only was there an apparent cure. Besides those operations, I have collected, from the experience of eminent surgeons, 703 cases, in which the breast was extirpated, and there were only twenty-two apparent cures. Still we must not regard every case where a second cancer tumour is discovered, as a relapse, because it is possible, of course, that they existed before the operation was performed, and that they were developed under the influence of causes similar to those which had occasioned the external disease. It is maintained by some persons, that the cancerous matter is softened, absorbed, carried into the circulation, and afterwards deposited in various organs; the absorption is not however sufficiently made out to be considered an established fact in science. If it be admitted as possible in external cancer, it must be equally possible when it is deposited in internal organs.

If cancer be reproduced after extirpation, it happens in different ways; sometimes the process of cicatrization is interrupted, fungous points or tubercles are developed, and a cancerous ulcer is soon produced. Sometimes the cicatrix is completed, after some time it is raised by a tumour, is destroyed, and the disease reappears. Sometimes it happens that the disease reappears in the lymphatic ganglia related to the part where the disease was originally seated. Those of the axilla, when the mammary gland has been affected, those of the pelvis, when the testicle has suffered. It may, however, occur in parts perfectly unconnected with the original seat of the disease.

In the relapses, the nature of the tissue sometimes changes, but usually it is the medullary structure. The interval comprised between the destruction of a cancerous tumour, and the reproduction, is very variable; sometimes it is very short, at others it is very long. But we know not why, no more than we know why in rabies so long an incubation sometimes happens.

*Exceptions?*—At a time when the impression is almost universal that cancer is a disease beyond the reach of art, and when, consequently, the disinclination to resort to extirpation of the morbid structure is daily increasing—when at the same time no one is disposed to deny that occasionally, though very unfrequently, cases occur in which reproduction does not happen—it is interesting to inquire whether we have any signs by which to distinguish a curable from an incurable cancer. Within a few years I have had opportunities of examining ten cases in which cancer commenced in the cutaneous integument, or portions of the mucous tissues nearly connected with it: one was a case in which the disease commenced in the perineum, and extended to the vagina; two were cases of cancer of the lip; one was a case of cancer on the penis; two were chimney-sweeps' cancers; in one, the disease had begun in the areolar tissue of the mammary gland; in one the rectum was the seat of the disease; and in one, the inside of the thigh. These cases were examined after death, three only dying from the immediate effects of the disease. In no one of these cases was a similar deposition found in any viscus. Supposing further experience confirm this as a common, though not a constant circumstance, it will still enable us to relax the rule which is now so stringently applied as to the unvarying fatality of the disease. It will be an evidence of the probability that cancer commencing in the cutaneous integument does not commonly, unless at an advanced period of its existence, pervade the system, so as to constitute a constitutional disease. Few surgeons of much experience have not seen cases in which cancer of the lip and cancer of the penis have been cured; and these cases no doubt depend upon the absence of constitutional infection. With regard to the other varieties, so much may be said, that though few, yet undoubted cases exist in which the disease has been extirpated, and no symptom of relapse has been manifested at the end of many years. In these cases, either no deposition of the carcinomatous product has occurred, the disease being local, or if deposited, its further development has been arrested. It is very important, therefore, to ascertain, by the aid of the microscope, and its general characters, the particular structure of each of the tumours which may be removed, as it may enable us to establish that certain other varieties of carcinomatous structures have little tendency, at least in the earlier periods of their existence, to infect the constitution generally, and that we may therefore recommend the removal as a probable curative means.

*Treatment.*—Two things must be necessary to cure cancer; the destruction of the cause, and the extirpation of the local disease. In surgery we extirpate the local disease, but we know that to obtain a cure both the local and general disease must be extirpated. If surgery obtains such inconsiderable success, it is because the extirpation of the local disease does not imply extirpation of the constitutional taint. In medicine, the success is even less, because neither the general nor local disease can in the present state of our knowledge be thus extinguished. The use of remedies has changed with the changing opinions as to the nature of the disease; when cancer was held to be a voracious animal, the hunger of this devouring vulture was appeased by placing upon the part slices of raw meat. The school of Broussais regarded it as inflammatory in its origin, and during its progress used the antiphlogistic treatment. Those who regarded it as a particular virus, employed such means as they conceived to be likely to neutralize that virus. These, and a great number of other fanciful means of treatment, have been employed and abandoned. In the present day, the medical treatment of cancer is directed to three main objects—to resolve the cancerous tumefaction, to alleviate the pain, and to change the constitution so far as to remedy the cachexia.

To attain the first object, mercurials, arsenicals, alkalies, iodine, and cicuta, hydrocyanic acid, iron, the Calendula officinalis, blood-letting, and emollients, have been extensively used, and many fancied cancerous tumours have seemed to yield under them.

To quiet pain, narcotics, such as opium, belladonna, aconite, and hemlock, have been much used; they may also be employed locally when the tumour is external. For many years, hemlock was conceived to have a specific action upon these tumours; indeed, the eulogiums of Storck led men to expect much from it. It has been much tried, but has never afforded any encouragement to hope that it has any other effect than that of lessening pain. Within a few years it has been very extensively employed in the practice of Recamier, in cases of cancer of the breast, the uterus, the testicle, the liver; and he believes with great success. He found the effects to be materially influenced by the quantity of food taken; if much food were taken, the action was null; if the patient were rigidly dieted, the action was very decided. His mode of administering the remedy was to take a dose morning and evening two hours before the first and the last meal, beginning with half a grain, and not exceeding six grains; this dose is continued for a fortnight, it is then increased to twelve grains each dose; this is continued for three or four weeks. Not more than one-third of the usual quantity of food should be taken, and it should be divided into three meals. Towards the end of the treatment the hemlock is gradually diminished, and the quantity of food as gradually increased.

M. Gama exhibited cicuta in combination with calomel—four parts of the former to one of the latter; one-grain pills are formed; at first, one is exhibited morning and evening, then two, increasing the dose one pill daily; in this way the dose has been increased to twenty-five, thirty, or even forty daily. As might be expected, the effects of this treatment are very decided; sometimes abundant salivation, at others purgation. If the patient be much pulled down by it, the treatment is suspended for a time. It is generally associated with antiphlogistics and the *cura famis.* In certain obstinate indurations, and certain unhealthy cancerous-looking ulcers of the head and face, it succeeds sometimes very well, but I have never seen any thing to assure me that it will cure true cancer tumours.

The means employed to remedy the cachexia are similar to what would be employed in cases of chronic inflammation; but they do not succeed; the food should be more vegetable than animal; irritating and stimulating substances should be avoided. Though the *cura famis* may succeed in cases of chronic inflammation it will not here, though it may be very useful as a principal or auxiliary remedy. Arsenic has been much used. Justamondy regarded it as a specific. Hill thought it a very valuable remedy. Biett and Thomson employ it in the form of an iodide. Allman has much used the iodide of potassium, and considers it very efficacious in cases of cancer of the face, the breast, and the uterus; he used it much externally; his formula was half a dram to a dram of the iodide, to an ounce and a half of lard. Mercury, Rust says, has succeeded in his experience in many cases of cancer of the lips and the tongue. In cases of true cancer these various means will, almost if not altogether, disappoint those who may employ them. And this impotence of all means of treatment, internally employed, induced men to attempt to remove the disease by means directly applied to it. These may be resolved into three; compression, caustics, and cutting instruments. It is desirable that you should perfectly comprehend that the action of these agents is strictly local, and that they do not strike at the root of the disease. Acting upon this opinion, some persons have rejected, in all cases, the assistance of surgery, and have urged that the patient should not be subjected to the pain and danger of a bloody operation, when this operation is at best only palliative. Besides, it has been observed in some cases that the wound resulting from ablation of the tumour becomes a cancerous ulcer, and the progress of the disease has appeared to be accelerated. Nevertheless, although I feel all the weight of these reasons, I think that the operation should in many cases be attempted, because cases of apparent cure do occur, and many cases of great relief. The extension of life which it procures is not dearly bought at the expense of the suffering of an operation. Of course, where the diseased mass cannot be completely circumscribed, an operation is contra-indicated. There are other circumstances which should make us pause; for instance, if hereditary transmission seemed probable; if the constitution seemed deeply impreseed. In such cases the disease would infallibly reappear and the suffering of the operation would probably hasten a fatal termination. It is contra-indicated when adhesions are so extensive as to render complete extirpation very improbable. There is another most important caution I have to give; do not operate, if you can avoid it, when the disease is making sensible progress. Its progress should be suspended. To this, some persons would demur; they would say, do not operate when its progress is suspended, because that suspension may last for years. But this confidence in the suspension is dangerous; often, a cancer, the progress of which has appeared to be arrested, suddenly lights up and proceeds rapidly. Nothing is, therefore, more reasonable than to profit by this period of repose to operate, instead of exposing the patient to the dangers of exacerbation. It is necessary to distinguish between the cancerous cachexia, which is a contra-indication, from that state of marasmus and feebleness, brought about by pain, which does not always contra-indicate operation. The general indications it is impossible to give; a calculation must be made between the strength of the patient and the dangers of the operation. It is also necessary to look at the age of the patient, for if, in the common course of things, he have not long to live, it is a matter of grave question whether a slight extension might not be too dearly purchased by operation. Still, advanced age does not absolutely prevent operation; and this is especially the case in those of the lip, tongue, and cheek.

*Compression.*—Dr. Young published his minutes of the cases of cancer and cancerous tendency, successfully treated by compression, in 1816. Sir Charles Bell was urged by the Governors of the Middlesex to give it a fair trial; he did, and reported that the compression of cancerous tumours, ulcerated or not, is injurious, and accelerates the progress of the disease. Fifteen years ago Recamier commenced extended experiments upon compression. In the first volume of his work, he says, a hundred patients presented themselves with cancer, sixteen seemed incurable, and were not treated; of the remaining eighty-four, thirty have been completely cured by compression; twenty-one have been improved; fifteen have been removed by ablation alone or combined with compression, and six by compression and cauterization; in the remaining twelve, the disease did not yield. That chronic tumours of the breast may be dissipated by carefully-made compression alone, or associated with blood-letting, is probable; that certain uterine tumours may yield to pressure I can believe; but I have never seen any thing to convince me that compression will cure a cancerous tumour. It is quite true that a cancerous tumour may apparently give way under well-applied pressure, but then a careful examination will show that the morbid structure still exists flattened, and the apparent diminution in the size of the organ is owing to an absorption of the cellular tissue, which always happens under pressure.

*Lond. Med. Gaz.*

---

*Contributions to the Pathology of new born Infants.* By Thomas H. Burgess, M. D.—Apoplexy and asphyxia in new born infants require such speedy and opposite treatment, that their diagnosis is extremely important. Dr. Burgess undertakes in this paper to illustrate apoplexy, intending on some future occasion to pursue the subject by observations on asphyxia. He details a case of birth in which there was some, but not unusual, delay, which would ordinarily lead to little fear of injury from mechanical causes.

The child cried loudly after the birth of the head, and again after the expulsion of the body; some mucoritus was discharged from the mouth; some food was taken, but within the first hour the hands were observed to be of a mottled, slaty colour. Dr. Burgess, when sent for, found the child of a blue colour all over, except the nose, which was pale and cold. The infant moaned feebly; the mouth was drawn aside; the fingers were clenched; and after a little blood had flowed from the left nostril, the child died.

Serous and bloody effusions were discovered within the skull and under the scalp, which the author minutely details.

A large quantity of serum was also effused into the pleural cavities of the chest.

After noticing the opinions of various writers, the author is inclined to think, that in this case the most probable cause of death was one suggested by M. Cruveilhier, that the uterine contractions exercised a fatal compression on the umbilical cord contained within the womb.

After recounting the morbid appearances commonly observed in similar cases, Dr. Burgess says, "none of these authors have noticed the sanguineous effusion *beneath* the arachnoid, and the engorgement of the choroid plexis, both of which conditions obtained in this instance."

The remedy, according to all authors, is blood-letting from the cord, which is directly opposed to the treatment required in asphyxia. The characteristic mark of infantine apoplexy is, according to M. Gardien, and the author of this paper, the livid hue of the body; while in asphyxia, the infant comes into the world, as Baudelocque says, "exsanguine." — *Lond. Med. Gaz.—Trans. Royal Med. and Chirurg. Society.*

